# RamanSPy: An Open-Source Python Package for Integrative Raman Spectroscopy Data Analysis

**DOI:** 10.1021/acs.analchem.4c00383

**Published:** 2024-05-15

**Authors:** Dimitar Georgiev, Simon Vilms Pedersen, Ruoxiao Xie, Álvaro Fernández-Galiana, Molly M. Stevens, Mauricio Barahona

**Affiliations:** † Department of Computing & UKRI Centre for Doctoral Training in AI for Healthcare, Imperial College London, London SW7 2AZ, United Kingdom; ‡ Department of Materials, Department of Bioengineering & Institute of Biomedical Engineering, Imperial College London, London SW7 2AZ, United Kingdom; § Department of Mathematics, Imperial College London, London SW7 2AZ, United Kingdom

## Abstract

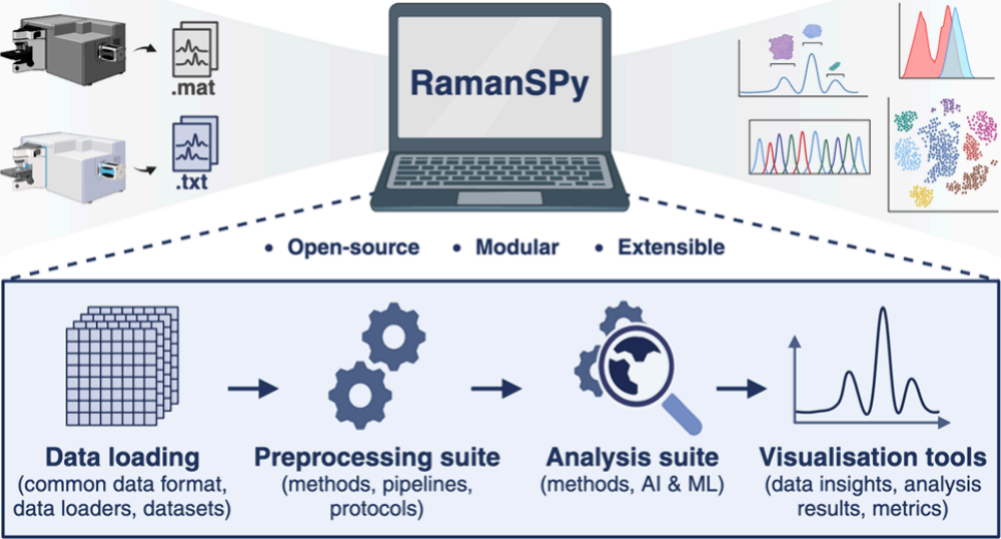

Raman spectroscopy is a nondestructive and label-free chemical analysis technique, which plays a key role in the analysis and discovery cycle of various branches of science. Nonetheless, progress in Raman spectroscopic analysis is still impeded by the lack of software, methodological and data standardization, and the ensuing fragmentation and lack of reproducibility of analysis workflows thereof. To address these issues, we introduce *RamanSPy*, an open-source Python package for Raman spectroscopic research and analysis. *RamanSPy* provides a comprehensive library of tools for spectroscopic analysis that supports day-to-day tasks, integrative analyses, the development of methods and protocols, and the integration of advanced data analytics. *RamanSPy* is modular and open source, not tied to a particular technology or data format, and can be readily interfaced with the burgeoning ecosystem for data science, statistical analysis, and machine learning in Python. *RamanSPy* is hosted at https://github.com/barahona-research-group/RamanSPy, supplemented with extended online documentation, available at https://ramanspy.readthedocs.io, that includes tutorials, example applications, and details about the real-world research applications presented in this paper.

## Introduction

Raman spectroscopy (RS) is a powerful sensing modality based on inelastic light scattering, which provides qualitative and quantitative chemical analyses with high sensitivity and specificity.^1^ RS yields a characterization of the vibrational profile of molecules, which can help elucidate the composition of chemical compounds, biological specimens, and materials.^2−4^ In contrast to most conventional technologies for (bio)chemical characterization (e.g., staining, different omics, fluorescence microscopy, and mass spectrometry), RS is both label-free and nondestructive, thereby allowing the acquisition of rich biological and chemical information without compromising the structural and functional integrity of the probed samples. This advantage has enabled a broad range of applications of RS across biomedical and pharmaceutical research,^3−15^ materials science,^16,17^ environmental science,^18,19^ and others.^20−22^

An area of topical interest is the frontier of Raman spectroscopy, chemometrics, and artificial intelligence (AI), with its promise of more autonomous, flexible, and data-driven RS analytics.^23−25^ There has been a recent surge in the adoption of AI methods in Raman-based research,^4^ with applications to RS now spanning domains as broad as the identification of pathogens and other microbes^26−29^; the characterization of chemicals, including minerals,^30^ pesticides,^31^ and other analytes^32,33^; the development of novel diagnostic platforms^34−37^; and the application of techniques from computer vision for denoising and super-resolution in Raman imaging.^38^

As new hardware, software, and data acquisition RS technologies continue to emerge,^39,40^ there is a pressing need for an integrated RS data analysis environment, which facilitates the development of pipelines, methods, and applications, and bolsters the use of RS in industry and research. Yet, the full deployment of RS and its capabilities is still hindered by practical factors stemming from the restrictive, functionally disparate, and highly encapsulated nature of current commercial software for RS data analysis. RS data analysis often operates within proprietary software environments and data formats, which have induced methodological inconsistencies and reduced cross-platform and integrative efforts, with growing concerns around reproducibility. These restrictions have also hampered the adoption of new AI technologies into the field.^41−45^ As a consequence, researchers increasingly resort to developing in-house scripts for RS analysis in Python,^46^ further adding to methodological fragmentation and lack of reproducibility.^47^

In response to these challenges, we have developed *RamanSPy*—a modular, open-source framework for integrated RamanSpectroscopy analytics in Python. *RamanSPy* is designed to systematize day-to-day workflows, enhance algorithmic development, integration, and reproducibility, and accelerate the adoption of novel AI technologies into the RS field. First, *RamanSPy* serves as a platform for general-purpose RS analytics supporting the RS data life cycle by providing a suite of ready-to-use modules for data loading, preprocessing, analysis, and visualization. By design, these functionalities are not tied to any specific technology or data type, thereby allowing integrative and transferable cross-platform analyses. Second, *RamanSPy* addresses challenges in data preprocessing by facilitating the compilation of reproducible pipelines to streamline and automatize preprocessing protocols. Third, *RamanSPy* helps bridge the gap between RS data and state-of-the-art AI technologies within the extensive machine learning (ML) ecosystem in Python. This is further complemented by direct access to relevant data sets, preprocessing protocols, and performance metrics.

## Experimental Section

### Cell Phenotyping via Spectral Unmixing

Several of our examples are based on data from Kallepitis et al.,^9^ which provided volumetric RS scans of four human leukemia monocytic (THP-1) cells. Here, we only used the first scan (scan *‘001’*). The raw THP-1 data used for the spectral unmixing procedure in Figure 2 was exported as *MATLAB* files from the *WITec Project FIVE* software. The *MATLAB* files were then loaded into *RamanSPy* followed by spectral preprocessing with a protocol consisting of (1) spectral cropping to the 700–1800 cm^–1^ region; (2) cosmic rays removal with the algorithm proposed in Whitaker and Hayes^48^; (3) denoising with a Savitzky–Golay filter^49^ of polynomial order 3 and kernel size 7; (4) baseline correction with asymmetric least squares^50^; and (5) Global MinMax normalization to the interval [0,1]. After preprocessing, we performed spectral unmixing in *RamanSPy* using N-FINDR^51^ (number of endmembers set to 5) and fully constrained least squares (FCLS).^52^ We concluded the analysis by visualizing the results corresponding to the top four endmembers.

### Preparing THP-1 Data for Deep-Learning Denoising

The denoising analysis on the data in Figure 4d,e was performed on the middle depth layer (fifth layer out of 10) of the THP-1 volumetric scan from Kallepitis et al.^9^ This layer consisted of a 40 × 40 image scan, i.e., 1600 spectra. To be consistent with the original paper,^38^ we conducted exactly the same preprocessing protocol described there. Namely, we utilized the *WITec Project FIVE* software to crop the data to the region 500–1800 cm^–1^, followed by baseline correction using the *“shape”* method with α = 500. To assess the performance of the deep-learning denoiser, we created “low signal-to-noise spectra” by adding Gaussian noise to the original spectra. Each spectrum was MinMax-normalized to the range 0–1, and Gaussian noise with a standard deviation σ = 0.15 was added. This resulted in spectra of similar noise levels to those in Horgan et al.^38^ These noisy samples were used as the input to the model, and the uncontaminated data was taken as ground-truth targets. We then MinMax-normalized each spectrum (both inputs and targets) and compared the performance of the neural network denoiser against six Savitzky–Golay filters.^49^ To make all models comparable, and to correct for potential artifacts of how the model was trained originally in Horgan et al.,^38^ all denoising metrics were computed after MinMax-normalizing the denoised outputs of each denoiser to the range 0–1 again.

### Computational Efficiency Analysis

We profiled the computational efficiency of a representative preprocessing protocol (*Pipeline I*), which consists of (1) spectral cropping to the 700–1800 cm^–1^ region; (2) cosmic rays removal with the algorithm proposed in Whitaker and Hayes^48^; (3) denoising with a Gaussian filter; (4) baseline correction with asymmetric least squares^50^; and (5) pixel-wise normalization based on setting the area under the curve to 1. We apply the pipeline to synthetic data of three sizes: 1000, 10,000, and 100,000 spectra, where each spectrum is generated by sampling 1500 values from a uniform distribution over [0, 1). We measured wall time on a MacBook Air laptop (Apple M2 chip, 8-core CPU, 10-core GPU, and 16-core Neural Engine).

## Results and Discussion

### *RamanSPy* as a Platform for General Raman Spectroscopy Analytics

**Figure 1:**
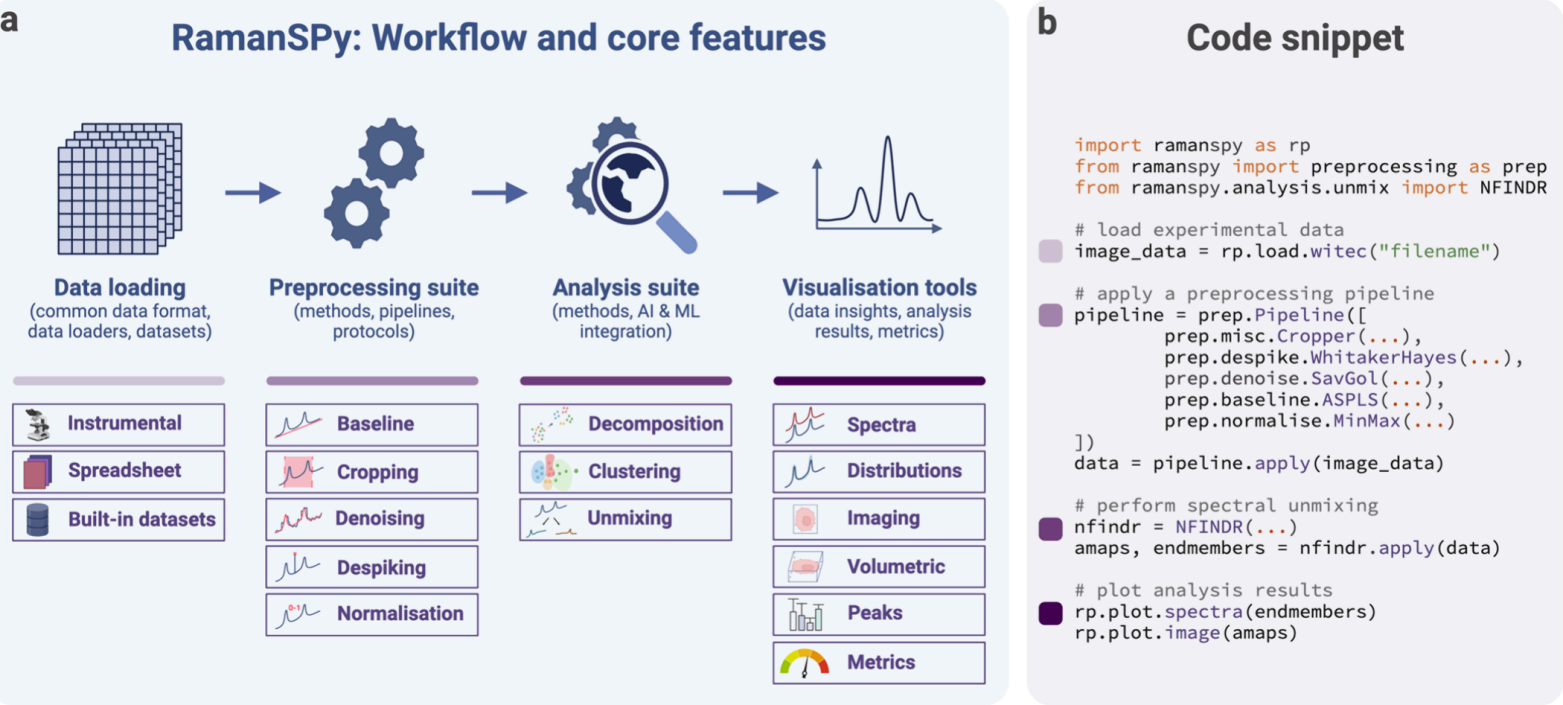
General Raman spectroscopy workflow and core features of *RamanSPy*. (a) *RamanSPy* supports the Raman spectroscopic data analysis life cycle via a modular, loosely coupled architecture. RS data is parsed to a common data representation format, which is interfaced with preprocessing, analysis, and visualization tools within *RamanSPy*. The core features of *RamanSPy* include a comprehensive library of standardized, simple-to-use procedures for data loading, preprocessing, analysis, and visualization. These modules are flexible and allow for the incorporation of further techniques and in-house methods. For complete information about the modules available in *RamanSPy*, refer to the documentation at https://ramanspy.readthedocs.io. (b) An example workflow use case in *RamanSPy*: Raman data is loaded, preprocessed, and analyzed in a few lines of code.

*RamanSPy* is based on a modular, object-oriented programming (OOP) infrastructure comprising a comprehensive collection of predefined tools for RS data analysis, which streamlines the analysis life cycle (Figure 1a) and reduces computational barriers to standard analyses (Figure 1b). The framework adopts a scalable array-based data representation that accommodates different spectroscopic modalities, including single-point spectra, Raman imaging data, and volumetric scans. Experimental data can be loaded through custom loaders built into *RamanSPy* or through standard tools available in Python. The data representation functions as a common data container that defines the interface between RS data management and manipulation within *RamanSPy*, facilitating the integrative analysis of data across setups and vendors when appropriate (e.g., measurements from patient samples acquired by different laboratories), independent of instrumental origin and acquisition modality.

On top of that, *RamanSPy* provides an extensive toolbox for preprocessing, analysis, and visualization. The preprocessing suite includes techniques for denoising, baseline correction, cosmic spike removal, normalization, and background subtraction, among others. Likewise, the analysis toolbox includes modules for decomposition (useful for dimensionality reduction), clustering, and spectral unmixing. *RamanSPy* also includes a set of data visualization tools, intended to facilitate routine visualization and exploratory analysis. All these modules are organized into a common class structure, which standardizes their application across projects and data sets to facilitate transferable analysis workflows. Note that this suite is highly flexible and designed to cater to a wide range of requirements, applications, and user profiles. On the one hand, *RamanSPy* enables the compilation of different workflows by using built-in tools as “building blocks” to streamline repetitive tasks and routine analyses. On the other hand, the package also retains the flexibility to develop and integrate custom methods as new “blocks” within the package for more advanced applications.

**Figure 2:**
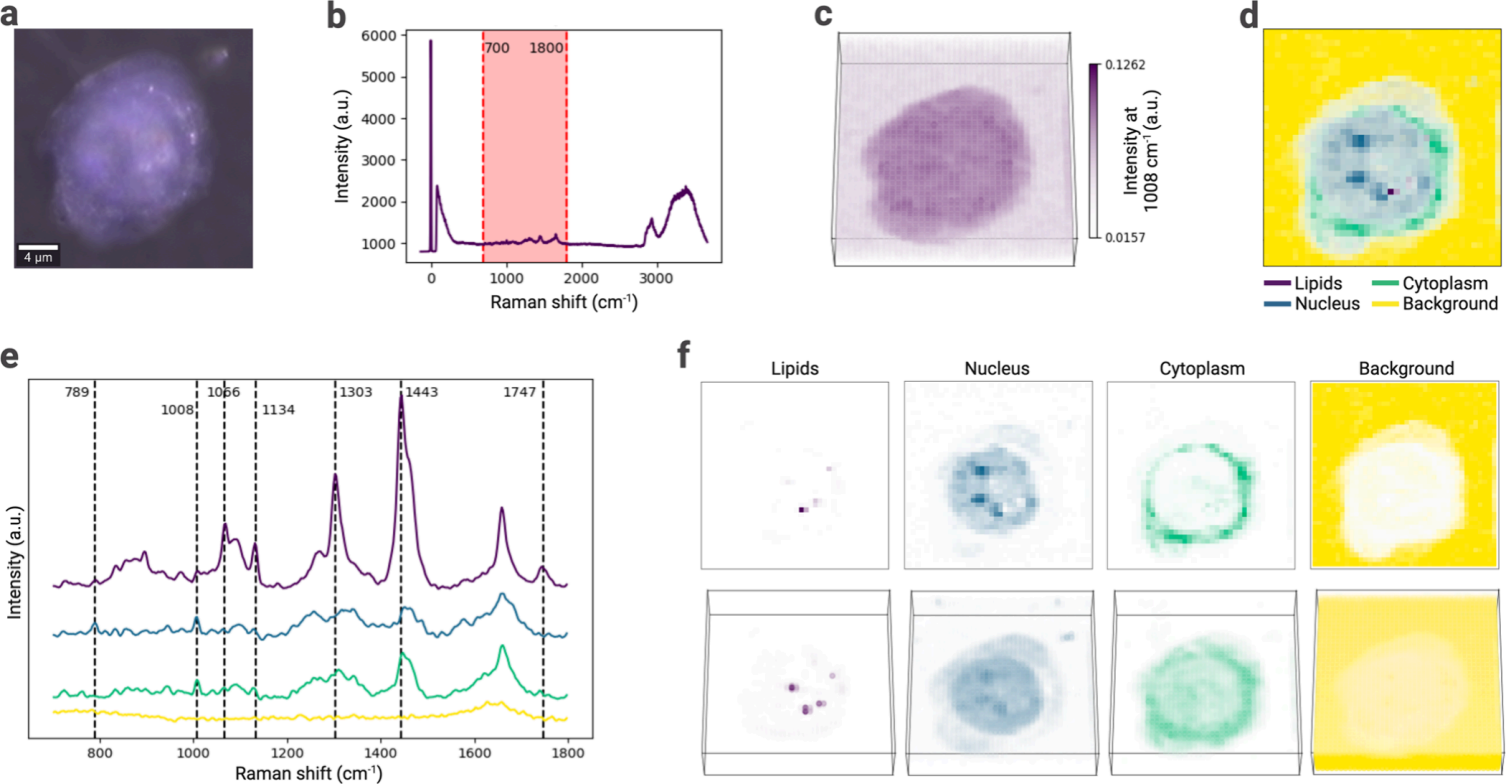
Morphological analysis of a THP-1 cell via spectral unmixing with *RamanSPy*. (a) Bright-field image of a THP-1 cell. The same cell was also imaged by Raman spectroscopy. Image and volumetric Raman data from Kallepitis et al.^9^ (b) An example spectrum from the raw volumetric Raman data (taken from the center region of the layer in d). The fingerprint region (700–1800 cm^–1^) shaded in red was used for the analysis. (c) Exploratory analysis: a volumetric slice across the 1008 cm^–1^ band (characteristic of proteins) after preprocessing. (d–f) Spectral unmixing analysis reveals the distribution of components within the cell: lipids (violet), nucleus (blue), cytoplasm (green), and background (yellow). (d) A merged reconstruction of the sixth depth layer (10 in total) of the THP-1 cell determined via spectral unmixing. (e) Four endmembers derived with N-FINDR^51^ characterized via peak assignment. (f) Fractional abundance maps calculated with FCLS^52^: (top), single slice (*z* = 6); (bottom), entire volume.

We showcase the core features of *RamanSPy* through real research application. In particular, we use *RamanSPy* to replicate the spectral unmixing analysis performed in Kallepitis et al.,^9^ where a volumetric Raman scan across a human leukemia monocytic (THP-1) cell was collected and analyzed using methods from chemometrics to investigate the cell phenotype in a label-free manner (Figure 2). We load the data using built-in data loaders and perform a spectral preprocessing protocol comprising spectral cropping to the fingerprint region (700–1800 cm^–1^), cosmic spike removal, denoising, baseline correction, and normalization. Using the visualization tools in the package, we inspect data quality (Figure 2b) and perform initial exploratory analysis by examining, e.g., data slices across wavenumber bands (Figure 2c). The analysis proceeds to spectral unmixing based on (i) N-FINDR^51^ for endmember detection and (ii) fully constrained least-squares (FCLS)^52^ for component quantification. This process is exploited to demix signal contributions from different cellular components and study their morphological organization within the THP-1 cell. Following the peak assignment and characterization in the original paper, we distinguish endmember components related to cytoplasm (band 1008 cm^–1^), nucleic acid (band 789 cm^–1^), lipids (bands 1066, 1134, 1303, 1443, and 1747 cm^–1^), and the background (Figure 2e). Finally, we produce fractional abundance reconstructions based on the extracted endmembers, which we can examine on a single-layer level and across the entire volume to localize cellular organelles within the cell (Figure 2f).

### *RamanSPy* Enables Automated Pipelining of Spectral Preprocessing Protocols

Experimental RS data is susceptible to nonspecific signal artifacts (e.g., cosmic rays, autofluorescence background, variability in instrumentation), which can severely affect downstream analyses. Preprocessing is therefore a critical step in any spectroscopic analysis workflow.^53,54^ Yet, due to a lack of standardization and frameworks that streamline the preprocessing of RS data,^41^ researchers tend to utilize variable preprocessing protocols, often dispersed across different software systems, thus affecting reproducibility and validation.^55,56^

**Figure 3:**
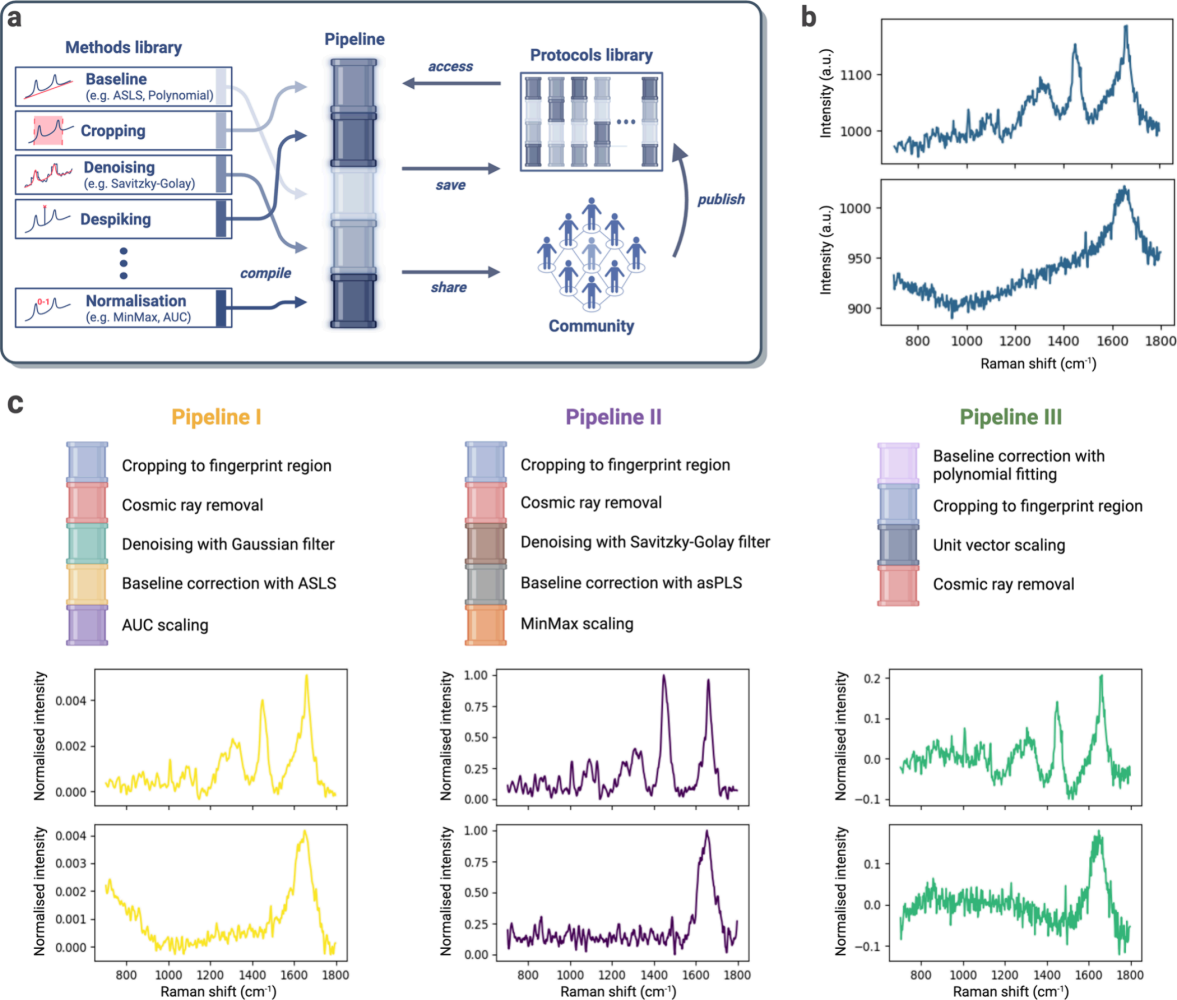
Spectral preprocessing pipelining in RamanSPy. (a) *RamanSPy* automates the construction, customization, and execution of multilayered preprocessing procedures via pipelining. Users can assemble built-in and in-house methods into complete preprocessing pipelines, which are fully compatible with data integrated within *RamanSPy* and can be saved, reused, and shared. *RamanSPy* also provides access to a library of preprocessing pipelines. (b) Two raw spectra from the THP-1 data from Kallepitis et al.^9^ are used to compare the effect of different preprocessing pipelines. (c) The results of three preprocessing pipelines built within *RamanSPy*, demonstrating the need for transparency and reproducibility. Note on preprocessing methods: fingerprint region is 700–1800 cm^–1^; ASLS—asymmetric least squares^50^; asPLS—adaptive smoothness penalized least squares^58^; AUC—area under the curve; cosmic rays removed with algorithm from Whitaker and Hayes.^48^

To facilitate the creation of reproducible protocols, *RamanSPy* incorporates a pipelining infrastructure, which systematizes the process of creating, customizing, and executing preprocessing pipelines (Figure 3a). Users can use a specialized class, which defines a generic, multilayered preprocessing procedure, to assemble pipelines from selected built-in preprocessing modules or other in-house methods. To reduce overhead, the constructed pipelines are designed to function exactly as any single method; i.e., they are fully compatible with the rest of the modules and data structures in the package. Furthermore, pipelines can be easily saved, reused, and shared (e.g., upon publication) to foster the development of a repository of preprocessing protocols. As a seed to this repository, *RamanSPy* provides a library of assembled preprocessing protocols (custom predefined, or adapted from the literature^57^), which users can access and exploit.

To illustrate the pipelining functionalities of *RamanSPy*, we constructed three representative preprocessing protocols. Within *RamanSPy*, this entails compiling selected methods in the desired order of execution and applying them out of the box to data loaded into the platform. We then use the three pipelines to preprocess Raman spectroscopic data from Kallepitis et al.^9^ (Figure 3b). Note how the three pipelines yield substantially different results (Figure 3c), reinforcing the importance of transparency and reproducibility in the selection of preprocessing protocols. We added these three pipelines to our repository of protocols in *RamanSPy*. As an indication of the computational efficiency of *RamanSPy* for such preprocessing tasks, we recorded the time it takes to preprocess spectra with *Pipeline I*, which represents a standard preprocessing protocol. Our experiments show that it takes 0.25 s to preprocess 1000 spectra with this protocol; 2.5 s for 10,000 spectra; and 20–25 s for 100,000 spectra (all computed on a standard laptop).

### *RamanSPy* Facilitates AI Integration and Validation of Next-Generation Raman Data Analytics

To help accelerate the adoption of AI technologies for RS analysis, *RamanSPy* is endowed with a permeable architecture that streamlines the interface between Raman spectroscopic data and the burgeoning ML ecosystem in Python. This is complemented by access to built-in data sets and performance metrics to further support the development and testing of new models and algorithms. We show below two examples of *RamanSPy’s* capabilities for ML integration and validation.

**Figure 4:**
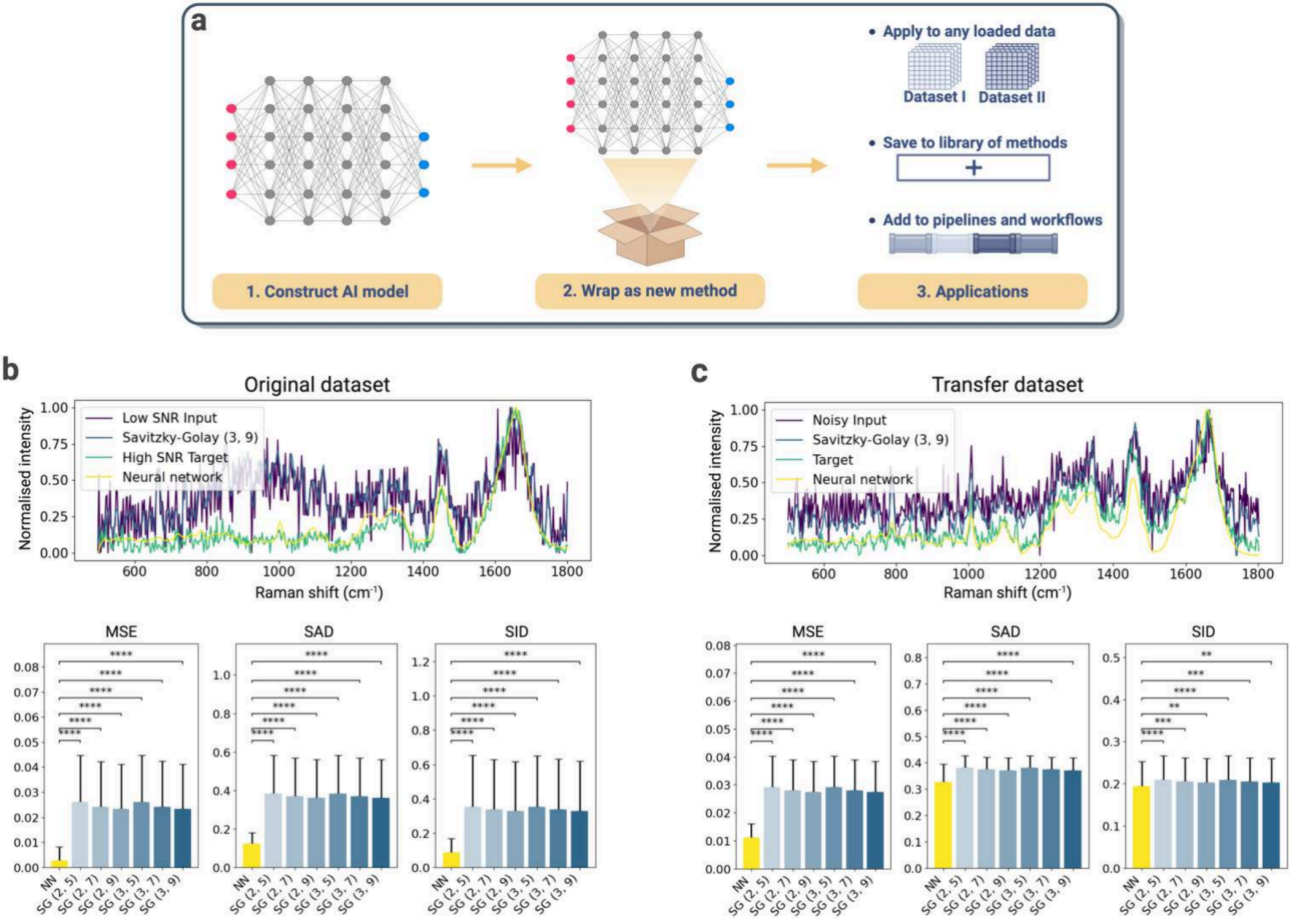
*RamanSPy* interfaces with AI/ML Python frameworks to create new methods for RS analysis. (a) *RamanSPy* allows users to incorporate AI/ML models seamlessly into pipelines created within the platform. (b) A pretrained 1D ResUNet deep-learning denoiser^38^ is integrated as a preprocessing module within *RamanSPy* to investigate its performance against the Savitzky–Golay (SG) filter.^49^ (Top) Denoising of a spectrum from Horgan et al.,^38^ where the low-SNR (purple) is the input and the high SNR (green) is the target. The data is denoised with an SG filter of polynomial order 3 and kernel size 9, SG(3, 9) (blue), and with the implemented deep-learning denoiser (yellow). (Bottom) The results on the test set from Horgan et al.^38^ (*n* = 12694) show that the deep-learning denoiser outperforms six SG filters across three performance metrics (MSE, SAD, SID). Error bars represent one standard deviation of the sample mean. Statistical significance measured with a two-sided Wilcoxon signed-rank test with adjustment for multiple comparisons based on Benjamini–Hochberg correction^59^ (*P < 0.05, **P < 0.01, ***P < 0.001, ****P < 0.0001). (c) Similar analysis on unseen data from Kallepitis et al.^9^ (*n* = 1600). The input (purple) corresponds to data contaminated with added noise, and the target (green) corresponds to the original data. In this case, the deep-learning denoiser only shows an improvement for MSE.

First, *RamanSPy* allows the seamless integration of standard Python AI/ML methods (e.g., from *scikit-learn*,^60^*PyTorch*,^61^ and *tensorflow*(62)) as tools for RS analysis (Figure 4a). As an illustration of how custom methods can be integrated into analysis pipelines within *RamanSPy*, we use our package to construct a deep-learning denoiser based on the one-dimensional ResUNet model—a fully convolutional UNet neural network with residual connections, presented in Horgan et al.^38^ To do this, we simply wrap within *RamanSPy* the pretrained neural network (trained on spectra from MDA-MB-231 breast cancer cells, available at https://github.com/conor-horgan/DeepeR) as a custom denoising method. Once wrapped, the denoiser is automatically compatible with the rest of *RamanSPy* and can be readily employed for different applications. For instance, we replicated the results in Horgan et al.^38^ and show in Figure 4b that the application of this deep-learning denoiser to the low signal-to-noise ratio (SNR) test set from Horgan et al.^38^ consistently outperforms the commonly used Savitzky–Golay filter.^58^ This is quantified by various metrics also coded within *RamanSPy* (e.g., mean squared error (MSE), spectral angle distance (SAD),^63^ and spectral information divergence (SID)^64^), which we use to measure the performance of each denoising method by comparing denoised signals against the provided high SNR data, which act as a reference.

Importantly, applying this pipeline to new data involves changing only the data source. Taking advantage of this transferability, we test the denoiser on unseen volumetric Raman data from another cell line (THP-1^9^), to which we added Gaussian noise. For these data, Figure 4c shows improved denoising performance according to the MSE metric, which is dependent on normalization and scale, but with lower significance according to scale-invariant metrics, also available in *RamanSPy*. This example emphasizes the sensitivity of algorithms to data shifts and the importance of incorporating robust validation criteria based on the unique requirements of each application. The collection of metrics that *RamanSPy* provides is intended to serve as a starting resource to test performance according to different objectives. We remark that this deep denoiser is used only to exemplify integration and testing capabilities of *RamanSPy* but is not included as a default denoiser in *RamanSPy*.

**Figure 5:**
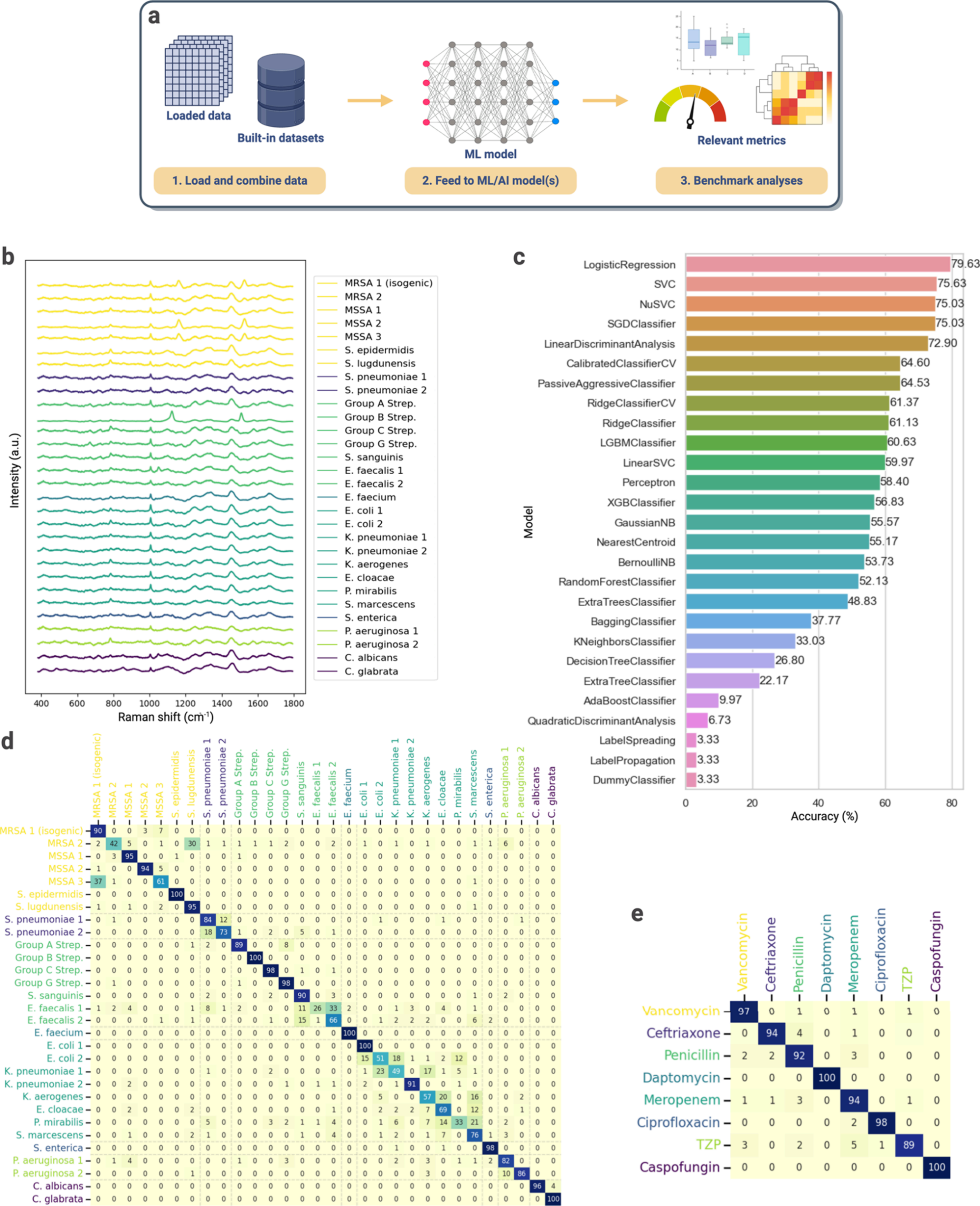
*RamanSPy* as a suite for algorithmic development. (a) Data representations in *RamanSPy* are compatible with the Python AI/ML ecosystem, allowing data flow from *RamanSPy* to *scikit-learn*,^60^*PyTorch*,^61^*tensorflow*,^62^ etc. *RamanSPy* is also equipped with standard data sets and relevant metrics to support model development and validation. (b–e) Benchmarking ML classification models on the task of bacteria identification using Raman spectra from Ho et al.^26^ (b) Mean Raman spectra of each bacterial species in the data set used for training. Spectra are min–max normalized to the range 0–1 for visualization purposes. (c) Benchmarking results of 28 ML models. The best accuracy was achieved using the logistic regression classifier. (d, e) Confusion matrices for the best species-level (d) and antibiotic-level (e) classifier with accuracies of 79.63 and 94.63%, respectively.

Second, the data management backbone of *RamanSPy* ensures a direct data flow to the rest of the Python ecosystem, i.e., data can be loaded, preprocessed, and analyzed in *RamanSPy* and then exported to conduct further modeling and analysis elsewhere (Figure 5a). As an example application, we perform AI-based bacteria identification using Raman measurements from 30 bacterial and yeast isolates as provided in Ho et al.^26^ (Figure 5b). After loading and visualizing the spectra with *RamanSPy*, we interface the data with the *lazypredict* Python package,^65^ which allows us to directly benchmark 28 ML classification models (including logistic regression, support vector machines, and decision trees) on the task of predicting the bacterial species from a given spectrum. The models were first trained on the fine-tuning data set (100 spectra per isolate) and then tested on the unseen test set of the same size. Our benchmarking analysis in Figure 5c finds logistic regression as the best-performing model, achieving a classification accuracy of 79.63% on the species-level classification task (Figure 5d) and 94.63% for antibiotic treatment classification (Figure 5e).

To further assist the process of testing and evaluating new computational approaches, *RamanSPy* provides access to a library of curated data sets for different tasks (e.g., classification, denoising, Raman imaging). With this library, we aim to seed the growth of a common repository of RS data sets that helps reduce barriers to data access, especially for ML teams with limited access to RS instruments.^24^ The data set library in *RamanSPy* already includes data loaders for Raman data from bacterial species,^26^ cell lines,^9,38^ COVID-19 samples,^66,67^ multi-instrument surface-enhanced Raman spectroscopy (SERS) measurements of adenine samples,^68^ wheat lines,^69^ and minerals^70^ and will continue to be expanded. Recognizing the potential benefits that synthetic and surrogate data sets can provide for the generation of controlled ground truths for algorithmic validation, we have also integrated the synthetic Raman data generator described in Georgiev et al.^71^ within *RamanSPy*.

## Conclusions

In this paper, we have introduced *RamanSPy*—a computational framework for integrative Raman spectroscopic data analysis aimed at overcoming some of the limitations of currently available commercial software tools in terms of accessibility, flexibility, reproducibility, and advanced data analysis capabilities. *RamanSPy* offers a comprehensive collection of tools for spectroscopic analysis designed to systematize the RS data analysis life cycle, reducing typical overheads of analysis workflows and improving methodological standardization. The package also lays the foundations of a common repository of standardized methods, protocols, and data sets, which users can readily access and exploit. Furthermore, *RamanSPy* is fully compatible with frameworks for data science and ML in Python, thereby facilitating the adoption and validation of advanced AI technologies for next-generation RS analysis. Lastly, we remark that, while our focus here has been on Raman spectroscopy and the unique requirements and challenges inherent to Raman data, many of the tools in *RamanSPy* (e.g., denoising and baseline correction) are of broad applicability to other spectroscopy techniques, including infrared (IR) and ultraviolet–visible (UV–vis) spectroscopy.

*RamanSPy* is fully open-source and disseminated under a permissive license that allows for unrestricted use, adaptation, and extension, including commercial purposes. We believe this will be critical for the continuous development of the platform and its adoption across different scientific domains, including biomedical research, chemistry, and materials science, among others. Future directions include the expansion of our suite of built-in methods, tools, and data sets; the incorporation of cutting-edge AI technologies into the framework as the field progresses; and the integration of the package into experimental setups and other software solutions.

## Supporting Information Available

Experimental materials and methods regarding data preparation, preprocessing, and analysis (PDF)
Set of features of *RamanSPy* (April 2024) (PDF)
Implementation of the *RamanSPy* package, as well as its core features and intended use (PDF)

## Notes

All data used in this article are previously published open-access data that have been deposited by the respective authors online. Instructions on how to access, download, and load the data sets provided in *RamanSPy* are available in the documentation at https://ramanspy.readthedocs.io/en/latest/datasets.html.

## Notes

The codebase of *RamanSPy* is open-source and hosted on GitHub at https://github.com/barahona-research-group/RamanSPy. The package can be installed via pip using *‘pip install ramanspy’*. Documentation, including detailed tutorials and examples, is available at https://ramanspy.readthedocs.io. The scripts used to produce the analysis results presented in this paper are also provided as executable Jupyter Notebook examples at https://github.com/barahona-research-group/RamanSPy/tree/3dd2c1e09420c5ac473a72ebd6ed06a91c30a85c/paper_reproducibility and as part of the documentation of *RamanSPy* at https://ramanspy.readthedocs.io/en/latest/auto_examples/index.html.
