# Supplementary material for: RamanSPy: An Open-Source Python Package for Integrative Raman Spectroscopy Data Analysis
Source: Anal Chem. 2024 May 15;96(21):8492–500. doi: 10.1021/acs.analchem.4c00383 (PMC11140669; doi:10.1021/acs.analchem.4c00383)
Supplement: Supplementary file 1 — ac4c00383_si_001.pdf [file ac4c00383_si_001.pdf]

Supporting Information

RamanSPy: An open-source Python package for integrative Raman spectroscopy data analysis

Dimitar Georgiev[†,‡], Simon Vilms Pedersen[‡,§], Ruoxiao Xie[‡], Álvaro Fernández-Galiana[‡], Molly M. Stevens[‡,*] and Mauricio Barahona[¶,*]

† *Department of Computing & UKRI Centre for Doctoral Training in AI for Healthcare, Imperial College London, London, United Kingdom, SW7 2AZ*
‡ *Department of Materials, Department of Bioengineering & Institute of Biomedical Engineering, Imperial College London, London, United Kingdom, SW7 2AZ*
¶ *Department of Mathematics, Imperial College London, London, United Kingdom, SW7 2AZ*
§ *Present address: SDU Biotechnology, Faculty of Engineering, University of Southern Denmark, Denmark, 5230*

* To whom correspondence should be addressed to: Molly M. Stevens (m.stevens@imperial.ac.uk) and Mauricio Barahona (m.barahona@imperial.ac.uk).

Abstract: This file contains additional experimental materials and methods regarding data preparation, preprocessing and analysis.

Cell phenotyping via spectral unmixing. Several of our examples are based on data from Kallepitis et al.[1] which provided volumetric RS scans across 4 distinct THP-1 cell lines. Here, we only used the first scan (scan *'001'*). The raw THP-1 data from Kallepitis et al.[1] used for the spectral unmixing procedure in Fig. 2 was re-exported as *MATLAB* files from the *WITec Project FIVE* software. The *MATLAB* files were then loaded into *RamanSPy* followed by spectral preprocessing with a protocol consisting of: (1) spectral cropping to the 700-1800cm^{-1} region; (2) cosmic rays removal with the algorithm in Whitaker and Hayes[2]; (3) denoising with a Savitzky-Golay filter[3] of polynomial order 3 and kernel size 7; (4) baseline correction with asymmetric least squares[4]; and (5) Global MinMax normalisation to the interval [0,1]. After preprocessing, we performed spectral unmixing in *RamanSPy* using N-FINDR[5] (number of endmembers set to 5) and FCLS[6]. We concluded the analysis by visualising the results corresponding to the top 4 endmembers.

Preparing THP-1 data for deep learning denoising. The denoising analysis on the data in Fig. 4d-e was performed on the middle depth layer (fifth layer out of 10) of the THP-1 volumetric scan from Kallepitis et al.[1]. This layer consisted of a 40x40 image scan, i.e., 1600 spectra. To be consistent with the original paper[7], we conducted exactly the same preprocessing protocol described there. Namely, we utilised the *WITec Project FIVE* software to crop the data to the region 500-1800cm^{-1}, followed by baseline correction using the *'shape'* method with α=500. To assess the performance of the deep learning denoiser, we created `low-SNR spectra' by adding Gaussian noise to the original spectra. Each spectrum was MinMax-normalised to the range 0-1 and Gaussian noise with a standard deviation σ=0.15 was added. This resulted in spectra of similar noise levels to those in Horgan et al.[7]. These noisy samples were used as the input to the model and the uncontaminated data was taken as ground-truth targets. We then MinMax-normalised each spectrum (both inputs and targets) and compared the performance of the neural network denoiser against six Savitzky-Golay filters[3]. To make all models comparable, and to correct for potential artefacts of how the model was trained originally in Horgan et al.[7], all denoising metrics were computed after MinMax-normalising the denoised outputs of each denoiser to the range 0-1 again.

Computational efficiency analysis. We profile the computational efficiency of a representative preprocessing protocol (*Pipeline I*), which consists of: (1) spectral cropping to the 700-1800cm^{-1} region; (2) cosmic rays removal with the algorithm in Whitaker and Hayes[2]; (3) denoising with a Gaussian filter; (4) baseline correction with asymmetric least squares[4]; and (5) pixel-wise normalisation based on setting the area under the curve to 1. We apply the pipeline to synthetic data of three sizes - 1000, 10000 and 100000 spectra, where each spectrum is generated by sampling 1500 values from a uniform distribution over [0, 1). We measure wall time on a MacBook Air laptop (Apple M2 chip, 8-core CPU, 10-core GPU, and 16-core Neural Engine).

REFERENCES

[1] Kallepitis, C.; Bergholt, M. S.; Mazo, M. M.; Leonardo, V.; Skaalure, S. C.; Maynard, S. A.; Stevens, M. M. Quantitative volumetric Raman imaging of three dimensional cell cultures. *Nature communications* 2017, 8, 1–9.

[2] Whitaker, D. A.; Hayes, K. A simple algorithm for despiking Raman spectra. *Chemometrics and Intelligent Laboratory Systems* 2018, 179, 82–84.

[3] Savitzky, A.; Golay, M. J. Smoothing and differentiation of data by simplified least squares procedures. *Analytical chemistry* 1964, 36, 1627–1639.

[4] Eilers, P. H.; Boelens, H. F. Baseline correction with asymmetric least squares smoothing. *Leiden University Medical Centre Report* 2005, 1, 5.

[5] Winter, M. E. N-FINDR: An algorithm for fast autonomous spectral end-member determination in hyperspectral data. *Imaging Spectrometry V.* 1999; pp 266–275.

[6] Heinz, D. C.; Chang, C.-I. Fully constrained least squares linear spectral mixture analysis method for material quantification in hyperspectral imagery. *IEEE transactions on geoscience and remote sensing* 2001, 39, 529–545.

[7] Horgan, C. C.; Jensen, M.; Nagelkerke, A.; St-Pierre, J.-P.; Vercauteren, T.; Stevens, M. M.; Bergholt, M. S. High-throughput molecular imaging via deep-learning-enabled Raman spectroscopy. *Analytical chemistry* 2021, 93, 15850–15860.
