# Supplementary material for: RamanSPy: An Open-Source Python Package for Integrative Raman Spectroscopy Data Analysis
Source: Anal Chem. 2024 May 15;96(21):8492–500. doi: 10.1021/acs.analchem.4c00383 (PMC11140669; doi:10.1021/acs.analchem.4c00383)
Supplement: Supplementary file 2 — ac4c00383_si_002.pdf [file ac4c00383_si_002.pdf]

Supporting Information

RamanSPy: An open-source Python package for integrative Raman spectroscopy data analysis

Dimitar Georgiev[†,‡], Simon Vilms Pedersen[‡,§], Ruoxiao Xie[‡], Álvaro Fernández-Galiana[‡], Molly M. Stevens[‡,*] and Mauricio Barahona[¶,*]

† *Department of Computing & UKRI Centre for Doctoral Training in AI for Healthcare, Imperial College London, London, United Kingdom, SW7 2AZ*
‡ *Department of Materials, Department of Bioengineering & Institute of Biomedical Engineering, Imperial College London, London, United Kingdom, SW7 2AZ*
¶ *Department of Mathematics, Imperial College London, London, United Kingdom, SW7 2AZ*
§ *Present address: SDU Biotechnology, Faculty of Engineering, University of Southern Denmark, Denmark, 5230*

* To whom correspondence should be addressed to: Molly M. Stevens (m.stevens@imperial.ac.uk) and Mauricio Barahona (m.barahona@imperial.ac.uk).

Abstract: This file contains a table summarising the set of features of RamanSPy (April 2024).

Table S1. A table summarizing the main features of RamanSPy (April 2024; continued on following pages).

DATA LOADING AND MANAGEMENT

Common data format
- Single spectra
- Raman line scans
- Raman imaging data
- Volumetric Raman imaging data
- Collections of the above

Data loaders
- Built-in data loaders
  - WITec Suite (WITec)
  - WiRE (Renishaw)
  - OceanView (Ocean Insight)
  - LabSpec (HORIBA)
- Interface for integration of new data loaders

Datasets
- Bacteria data[1]
- Volumetric THP-1 cell data[2]
- MDA-MB-231 cells data[3]
- COVID-19 data[4-5]
- Adenine data[6]
- Wheat lines data[7]
- RRUFF data[8]
- Synthetic data generator[9]

PREPROCESSING

Built-in preprocessing methods

- Miscellaneous
  - Spectral cropping
  - Subtracting a fixed background signal
- Cosmic rays removal
  - Whitaker-Hayes despiking[10]
- Denoising
  - Savitzky-Golay filtering[11]
  - Discrete Penalised Least Squares (a.k.a Whittaker–Henderson smoothing)[12]
  - Kernel/window smoothers
  - Gaussian denoising
- Baseline correction
  - Least squares:
    - Asymmetric Least Squares (AsLS)[13]
    - Improved Asymmetric Least Squares (IAsLS)[14]
    - Adaptive Iteratively Reweighted Penalized Least Squares (airPLS)[15]
    - Asymmetrically Reweighted Penalized Least Squares (arPLS)[16]
    - Doubly Reweighted Penalized Least Squares (drPLS)[17]
    - Improved Asymmetrically Reweighted Penalized Least Squares (IarPLS)[18]
    - Adaptive Smoothness Penalized Least Squares (asPLS)[19]
  - Polynomial fitting:
    - Polynomial fitting
    - Modified polynomial fitting[20-21]
    - Penalised polynomial fitting[22]
    - Improved modified polynomial fitting[23]
  - Other:
    - Goldindec method[24]
    - Iterative Reweighted Spline Quantile Regression (IRSQR)[25]
    - Corner Cutting[26]
    - Fully automatic baseline correction (FABC)[27]
- Normalisation/Scaling
  - Vector normalisation
  - Min-max normalisation
  - Max intensity normalisation
  - Area-under-the-curve normalisation
- Interface for integration of custom algorithms

Preprocessing pipelines

- Built-in protocols
  - The 3 preprocessing protocols used in Fig. 3 in main text
  - A basic preprocessing protocol based on the one adopted in Bergholt et al (2016)[28]
- Interface for the development of custom pipelines

ANALYSIS	**Built-in analysis methods**<br>• Decomposition<br>o PCA<br>o NMF<br>o ICA<br>• Clustering<br>o KMeans<br>• Spectral unmixing<br>o N-FINDR[29]<br>o VCA[30]<br>o NNLS[31]<br>o FCLS[32]<br><br>**Metrics**<br>• MAE<br>• MSE<br>• RMSE<br>• SAD[33]<br>• SID[34]<br><br>**Interface with common AI & ML frameworks**
DATA VISUALISATION	**Spectra**<br>• Collections of spectra<br>• Distributions of spectra<br>• Peak finding<br><br>**Raman imaging data**<br>• Image slices across specific spectral bands<br>• Spectra at specific locations<br><br>**Volumetric Raman imaging data**<br>• Volumetric slices across specific spectral bands<br>• Image slices of specific z-slices<br>• Spectra at specific locations<br><br>**Peak distributions**

REFERENCES

[1] Ho, C.-S.; Jean, N.; Hogan, C. A.; Blackmon, L.; Jeffrey, S. S.; Holodniy, M.; Banaei, N.; Saleh, A. A.; Ermon, S.; Dionne, J. Rapid identification of pathogenic bacteria using Raman spectroscopy and deep learning. *Nature communications* **2019**, 10, 1–8.

[2] Kallepitis, C.; Bergholt, M. S.; Mazo, M. M.; Leonardo, V.; Skaalure, S. C.; Maynard, S. A.; Stevens, M. M. Quantitative volumetric Raman imaging of three dimensional cell cultures. *Nature communications* **2017**, 8, 1–9.

[3] Horgan, C. C.; Jensen, M.; Nagelkerke, A.; St-Pierre, J.-P.; Vercauteren, T.; Stevens, M. M.; Bergholt, M. S. High-throughput molecular imaging via deep-learning-enabled Raman spectroscopy. *ACS Analytical Chemistry* **2021**, 93, 15850–15860.

[4] Yin, G.; Li, L.; Lu, S.; Yin, Y.; Su, Y.; Zeng, Y.; Luo, M.; Ma, M.; Zhou, H.; Orlandini, L., et al. An efficient primary screening of COVID-19 by serum Raman spectroscopy. *Journal of Raman Spectroscopy* **2021**, 52, 949–958.

[5] Yin, G.; Li, L.; Lu, S.; Yin, Y.; Su, Y.; Zeng, Y.; Luo, M.; Ma, M.; Zhou, H.; Yao, D.; Liu, G.; Lang, J. Data and code on serum Raman spectroscopy as an efficient primary screening of coronavirus disease in 2019 (COVID-19). *Figshare* **2020**, DOI: 10.6084/m9.figshare.12159924.v1.

[6] Fornasaro, S.; Alsamad, F.; Baia, M.; Batista de Carvalho, L. A.; Beleites, C.; Byrne, H. J.; Chiadò, A.; Chis, M.; Chisanga, M.; Daniel, A., et al. Surface enhanced Raman spectroscopy for quantitative analysis: results of a large-scale European multi-instrument interlaboratory study. *ACS Analytical Chemistry* **2020**, 92, 4053–4064.

[7] Sen, A.; Kecoglu, I.; Ahmed, M.; Parlatan, U.; Unlu, M. Differentiation of advanced generation mutant wheat lines: Conventional techniques versus Raman spectroscopy. *Frontiers in Plant Science* **2023**, 14.

[8] Lafuente, B.; Downs, R. T.; Yang, H.; Stone, N. Highlights in mineralogical crystallography; *De Gruyter*, **2015**; pp 1–30.

[9] Georgiev, D.; Fernández-Galiana, A.; Pedersen, S.V.; Papadopoulos, G.; Xie, R.; Stevens, M.M; Barahona, M. Hyperspectral unmixing for Raman spectroscopy via physics-constrained autoencoders. *arXiv preprint* **2024**, arXiv:2403.04526.

[10] Whitaker, D. A.; Hayes, K. A simple algorithm for despiking Raman spectra. *Chemometrics and Intelligent Laboratory Systems* **2018**, 179, 82–84.

[11] Savitzky, A.; Golay, M. J. Smoothing and differentiation of data by simplified least squares procedures. *ACS Analytical Chemistry* **1964**, 36, 1627–1639.

[12] Eilers, P.H. A perfect smoother. *Analytical chemistry* **2003**, 75(14), 3631-3636.

[13] Eilers, P. H.; Boelens, H. F. Baseline correction with asymmetric least squares smoothing. *Leiden University Medical Centre Report* **2005**, 1, 5.

[14] He, S.; Zhang, W.; Liu, L.; Huang, Y.; He, J.; Xie, W.; Wu, P; Du, C. Baseline correction for Raman spectra using an improved asymmetric least squares method. *Analytical Methods* **2014**, 6(12), 4402-4407.

[15] Zhang, Z.M.; Chen, S.; Liang, Y.Z. Baseline correction using adaptive iteratively reweighted penalized least squares. *Analyst* **2010**, 135(5), 1138-1146.

[16] Baek, S.J.; Park, A.; Ahn, Y.J.; Choo, J., **2015**. Baseline correction using asymmetrically reweighted penalized least squares smoothing. *Analyst*, 140(1), 250-257.

[17] Xu, D.; Liu, S.; Cai, Y.; Yang, C. Baseline correction method based on doubly reweighted penalized least squares. *Applied optics* **2019**, 58(14), 3913-3920.

[18] Ye, J.; Tian, Z.; Wei, H.; Li, Y. Baseline correction method based on improved asymmetrically reweighted penalized least squares for the Raman spectrum. *Applied Optics* **2020**, 59(34), 10933-10943.

[19] Zhang, F.; Tang, X.; Tong, A.; Wang, B.; Wang, J.; Lv, Y.; Tang, C.; Wang, J. Baseline correction for infrared spectra using adaptive smoothness parameter penalized least squares method. *Spectroscopy Letters* **2020**, 53(3), pp.222-233.

[20] Gan, F.; Ruan, G.; Mo, J. Baseline correction by improved iterative polynomial fitting with automatic threshold. *Chemometrics and Intelligent Laboratory Systems* **2006**, 82(1-2), 59-65.

[21] Lieber, C.A.; Mahadevan-Jansen, A. Automated method for subtraction of fluorescence from biological Raman spectra. *Applied spectroscopy* **2003**, 57(11), 1363-1367.

[22] Mazet, V.; Carteret, C.; Brie, D.; Idier, J.; Humbert, B. Background removal from spectra by designing and minimising a non-quadratic cost function. *Chemometrics and intelligent laboratory systems* **2005** 76(2),121-133.

[23] Zhao, J.; Lui, H.; McLean, D.I.; Zeng, H. Automated autofluorescence background subtraction algorithm for biomedical Raman spectroscopy. *Applied spectroscopy* **2007**, 61(11), 1225-1232.

[24] Liu, J.; Sun, J.; Huang, X.; Li, G.; Liu, B. Goldindec: a novel algorithm for Raman spectrum baseline correction. *Applied spectroscopy* **2015**, 69(7), 834-842.

[25] Han, Q.; Peng, S.; Xie, Q.; Wu, Y.; Zhang, G. Iterative reweighted quantile regression using augmented Lagrangian optimization for baseline correction. *International Conference on Information Science and Control Engineering* **2018**, 280-284.

[26] Liu, Y.; Zhou, X.; Yu, Y. A concise iterative method using the Bezier technique for baseline construction. *Analyst* **2015**, 140(23), 7984-7996.

[27] Cobas, J.C.; Bernstein, M.A.; Martín-Pastor, M.; Tahoces, P.G. A new general-purpose fully automatic baseline-correction procedure for 1D and 2D NMR data. *Journal of Magnetic Resonance* **2006**, 183(1), 145-151.

[28] Bergholt, M. S.; St-Pierre, J.-P.; Offeddu, G. S.; Parmar, P. A.; Albro, M. B.; Puetzer, J. L.; Oyen, M. L.; Stevens, M. M. Raman spectroscopy reveals new insights into the zonal organization of native and tissue-engineered articular cartilage. *ACS Central Science* **2016**, 2, 885–895.

[29] Winter, M. E. N-FINDR: An algorithm for fast autonomous spectral end-member determination in hyperspectral data. *Imaging Spectrometry V* **1999**, 266–275.

[30] Nascimento, J.M.; Dias, J.M. Vertex component analysis: A fast algorithm to unmix hyperspectral data. *IEEE transactions on Geoscience and Remote Sensing* **2005**, 43(4), 898-910.

[31] Lawson, C.L.; Hanson, R.J. Solving least squares problems. *Society for Industrial and Applied Mathematics* **1995**.

[32] Heinz, D. C.; Chang, C.-I. Fully constrained least squares linear spectral mixture analysis method for material quantification in hyperspectral imagery. *IEEE transactions on geoscience and remote sensing* **2001**, 39, 529–545.

[33] Kruse, F. A.; Lefkoff, A.; Boardman, J.; Heidebrecht, K.; Shapiro, A.; Barloon, P.; Goetz, A. The spectral image processing system (SIPS)—interactive visualization and analysis of imaging spectrometer data. *Remote sensing of environment* **1993**, 44, 145–163.

[34] Chang, C.-I. Spectral information divergence for hyperspectral image analysis. *IEEE International Geoscience and Remote Sensing Symposium* **1999**, 509–511.
