# Supplementary material for: RamanSPy: An Open-Source Python Package for Integrative Raman Spectroscopy Data Analysis
Source: Anal Chem. 2024 May 15;96(21):8492–500. doi: 10.1021/acs.analchem.4c00383 (PMC11140669; doi:10.1021/acs.analchem.4c00383)
Supplement: Supplementary file 3 — ac4c00383_si_003.pdf [file ac4c00383_si_003.pdf]

Supporting Information

RamanSPy: An open-source Python package for integrative Raman spectroscopy data analysis

Dimitar Georgiev[†,‡], Simon Vilms Pedersen[‡,§], Ruoxiao Xie[‡], Álvaro Fernández-Galiana[‡], Molly M. Stevens[‡,*] and Mauricio Barahona[¶,*]

† *Department of Computing & UKRI Centre for Doctoral Training in AI for Healthcare, Imperial College London, London, United Kingdom, SW7 2AZ*
‡ *Department of Materials, Department of Bioengineering & Institute of Biomedical Engineering, Imperial College London, London, United Kingdom, SW7 2AZ*
¶ *Department of Mathematics, Imperial College London, London, United Kingdom, SW7 2AZ*
§ *Present address: SDU Biotechnology, Faculty of Engineering, University of Southern Denmark, Denmark, 5230*

* To whom correspondence should be addressed to: Molly M. Stevens (m.stevens@imperial.ac.uk) and Mauricio Barahona (m.barahona@imperial.ac.uk).

Abstract: This file contains additional information about the implementation of the RamanSPy package, its core features and intended use. Discussions are complemented by code snippets showcasing how to access and use the package's features.

INSTALLATION

RamanSPy has been deposited in the Python Package Index (https://pypi.org/project/ramanspy) and can thus be directly installed via the common package installer *pip* for Python:

```
pip install ramanspy
```

To access the functionalities of the package after installation, users only need to import *RamanSPy* in their Python scripts. One can import the whole package:

```
import ramanspy
# or import ramanspy as rp
```

or individual modules or methods:

```
# individual modules
from ramanspy import load , preprocessing

# individual methods
from ramanspy.analysis.unmix import NFINDR
```

CORE FUNCTIONALITIES

Data management. Data in *RamanSPy* is represented by a set of custom data container classes based on scalable, computationally efficient array programming based on *numpy*[1], which correspond to different spectroscopic modalities. This includes the generic *SpectralContainer* class, as well as the more specialised *Spectrum*, *SpectralImage* and *SpectralVolume* classes representing single-point spectra (1D), imaging data (3D), volumetric data (4D) respectively. These classes define data-specific information and behaviour in the background to allow a smooth, user-friendly experience, regardless of the data of interest.

The containers can be initialised by providing the corresponding intensity data, the spectral axis (in cm^{-1}) and other relevant (meta) data. For instance:

```
raman_spectrum = ramanspy.Spectrum(intensity_data, spectral_axis,
*args, **kwargs)
raman_image = ramanspy.SpectralImage(intensity_data, spectral_axis,
*args, **kwargs)
```

Once created, data containers can be manipulated, visualised, saved and loaded as needed using the built-in tools in *RamanSPy*.

Note that for the most part, users would not need to manually populate these containers. Instead, they can take advantage of the data loading functionalities that *RamanSPy* provides.

Data loading. To support data loading, *RamanSPy* offers easy-to-use data loaders compatible with experimental Raman spectroscopic data from a range of instrumental vendors in the area. These loaders - available within *ramanspy.load* - automatically parse relevant data files and return the appropriate spectral container. As an example, users can load *MATLAB* files exported from *WITec's Project FOUR/FIVE* software using the following command:

```
raman_object = ramanspy.load.witec(<PATH>)
```

A full list of the data loaders built into *RamanSPy* is available as part of the documentation of the package at https://ramanspy.readthedocs.io/en/latest/loading.html.

Raman data can also be loaded via established data-loading tools in Python. For instance, one can use *pandas*[2] to load a spectrum from a *.csv* file with two columns storing the intensity data and the spectral axis by using:

```
import pandas as pd

data = pd.read_csv(csv_filename)
raman_spectrum = ramanspy.Spectrum(data["<intensity_column>"], data["<axis_column>"])
```

Spectral preprocessing. Preprocessing logic in *RamanSPy* is defined by the *PreprocessingStep* class, which defines most of the necessary preprocessing infrastructure in the background to ensure a smooth, data-agnostic experience via a single point of contact specified through their *apply()* method.

Yet, as with data loading, for the most part, users are not expected to use this class to manually implement and optimise such preprocessing methods themselves. Instead, the *RamanSPy* package provides a comprehensive toolbox of ready-to-use preprocessing methods, which users can access, customise and employ to compile a wide variety of preprocessing procedures. These preprocessing procedures are given as predefined classes within *ramanspy.preprocessing* which extend the *PreprocessingStep* class. To use these built-in methods, users need to create an instance of the selected technique. For instance:

```
denoiser = ramanspy.preprocessing.denoise.SavGol(*args, **kwargs)
baseline_corrector = ramanspy.preprocessing.baseline.ASLS(*args, **kwargs)
normaliser = ramanspy.preprocessing.normalise.MaxIntensity(*args, **kwargs)
```

Note that *RamanSPy* offers full control over relevant parameters, which can be supplied during initialisation via the **args* and ***kwargs* arguments.

As the methods inherit all operational logic defined within the parent *PreprocessingStep* class, they can be directly accessed and used on any data loaded in the framework through their *apply()* method:

```
preprocessesd_objects = denoiser.apply(<spectral object or collection of spectral objects>)
preprocessesd_objects = baseline_corrector.apply(<spectral object or collection of spectral objects>)
```

A full list of the methods for spectral preprocessing built into *RamanSPy* is available as part of the documentation of the package at https://ramanspy.readthedocs.io/en/latest/preprocessing.html.

If needed, users can also incorporate in-house methods into *RamanSPy* by wrapping the corresponding preprocessing logic into instances of the *PreprocessingStep* class. This can be done as follows:

```
def preprocessing_func(intensity_data, spectral_axis, *args, **kwargs):
    # Preprocess intensity_data and spectral_axis
    ...
    return updated_intensity_data, updated_spectral_axis

# wrapping the function together with the relevant *args and **kwargs
custom_preprocessing_method = ramanspy.preprocessing.PreprocessingStep(preprocessing_func, *args, **kwargs)
```

Then, the custom preprocessing method is fully compatible with the rest of *RamanSPy's* functionalities and out-of-the-box applicable to any data integrated within the package via its *apply()* method:

```
custom_preprocessing_method.apply(<spectral object or collection of spectral objects>)
```

Note that this class structure implies that these instances can then be saved (e.g. as *pickle* files) and, therefore, reused and shared as required afterwards.

Spectral analysis. As with preprocessing classes, users can access any built-in analysis method (available within the *ramanspy.analysis* sub-module) by creating an object instance of the corresponding class as follows:

```
nmf = ramanspy.analysis.decompose.NMF(*args, **kwargs)
kmeans = ramanspy.analysis.cluster.KMeans(*args, **kwargs)
unmixer = ramanspy.analysis.unmix.NFINDR(*args, **kwargs)
```

Once created, instances can be similarly accessed via their *apply()* method on any data loaded in *RamanSPy*.

```
cluster_maps, cluster_centres = kmeans.apply(<spectral object or collection of spectral objects>)
abundance_fractions, endmemebrs = unmixer.apply(<spectral object or collection of spectral objects>)
```

A full list of the methods for spectral analysis built into *RamanSPy* is available as part of the documentation of the package at https://ramanspy.readthedocs.io/en/latest/analysis.html.

Visualisation. The *RamanSPy* package also provides various visualisation tools available within the *ramanspy.plot* sub-module. As an example, one can plot spectra using the *spectra()* function:

```
ramanspy.plot.spectra(<spectra or collection of spectra>)
ramanspy.plot.show()  # or plt.show() after import matplotlib.pyplot as plt
```

Note that these functions are highly customisable. This can be done by providing relevant parameters to control the plot generation, as well as through *matplotlib's* customisation workflow.

```
import matplotlib.pyplot as plt

plt.figure(figsize = (5, 5))
ax = ramanspy.plot.spectra(<spectra or collection of spectra>, title="<str>", label="<str or list[str]>")
ax.set_ylabel("<str>")  # adding a label to the y-axis
plt.show()  # or ramanspy.plot.show()
```

A full list of the methods for data visualisation built into *RamanSPy* is available as part of the documentation of the package at https://ramanspy.readthedocs.io/en/latest/plot.html.

PREPROCESSING PIPELINES

Pipelining behaviour is defined by the *Pipeline* class in *RamanSPy*, which ensures that pipelines are accessible, simple-to-use and fully compatible with the rest of *RamanSPy*.

Creating a custom preprocessing pipeline. To assemble a preprocessing pipeline, one simply needs to stack relevant methods (built-in or custom) into the intended order of execution. For instance:

```
preprocessing_pipeline = ramanspy.preprocessing.Pipeline([
```

```
    ramanspy.preprocessing.denoise.SavGol(*args, **kwargs),
    ramanspy.preprocessing.baseline.ASLS(*args, **kwargs),
    ramanspy.preprocessing.normalise.MaxIntensity(*args, **kwargs),
    custom_preprocessing_method(*args, **kwargs)   # custom in-house
method
])
```

Constructed pipelines can then be applied exactly as single methods via their *apply()* method to any data loaded within *RamanSPy*.

```
preprocessesd_objects = preprocessing_pipeline.apply(<spectral object
or collection of spectral objects>)
```

As pipelines in *RamanSPy* are objects, they can also be directly saved in a convenient file format, such as *pickle* files. As such, they can then be reloaded, reused and shared as needed.

Access a predefined preprocessing pipeline. *RamanSPy* also provides a collection of built-in preprocessing pipelines. To access them, one can select the desired protocol from *ramanspy.preprocessing.protocols* as follows:

```
preprocessing_pipeline = ramanspy.preprocessing.protocols.PROTOCOL_X
```

A pre-defined *Pipeline* instance will be returned, which can similarly be employed directly through its *apply()* method.

A full list of the protocols for spectral preprocessing built into *RamanSPy* is available as part of the documentation of the package at https://ramanspy.readthedocs.io/en/latest/preprocessing.html#established-protocols.

AI INTEGRATION

Integrate AI methods into RamanSPy. To integrate new techniques for spectral preprocessing and analysis, users can take advantage of the extensible architecture of *RamanSPy* and wrap models and algorithms into custom classes. For instance, one can create a new denoiser method based on the *PyTorch*[3] model for denoising from Horgan et al.[4] by simply creating a function, which defines how the model can be used to preprocess a generic intensity data array, and then wrapping the method within a *PreprocessingStep* instance.

```
def nn_preprocesing(intensity_data, wavenumber_axis):
    intensity_data = v.reshape(-1, intensity_data.shape[-1])
    output                                                        =
model(torch.Tensor(intensity_data).unsqueeze(1)).cpu().detach().numpy
()
    output = np.squeeze(output).reshape(intensity_data.shape)
    return output, wavenumber_axis

nn_denoiser                                                       =
ramanspy.preprocessing.PreprocessingStep(nn_preprocesing)
```

Integrated methods are automatically rendered fully compatible with the rest of *RamanSPy's* functionalities in the background, so one can simply use the *apply()* method of the constructed denoiser to preprocess any data loaded within *RamanSPy* as any built-in preprocessing class.

Export data from RamanSPy to AI frameworks. The data management core of *RamanSPy* allows a direct interface with the entire Python ecosystem, including frameworks for statistical modelling, machine learning and deep learning. To do that, users can simply feed relevant data from *RamanSPy* to functions and tools they want to use elsewhere. For instance, one can pass the intensity data stored in a spectral container to a specific model from the *scikit-learn*[5] framework for statistical and ML modelling directly via their *fit()* method:

```
model.fit(spectral_container.spectral_data)
```

DATASETS

To access the Raman spectroscopic datasets available in *RamanSPy*, users can employ custom data-loading methods built into *RamanSPy* under *ramanspy.datasets*. These would automatically parse the relevant data into the corresponding spectral container. For instance, one can load the bacteria data from Ho et al.[6] using the following function:

```
data_container,labels = ramanspy.datasets.bacteria(dataset="train",<PATH>)
```

Note that, depending on where each dataset was deposited and the license it was deposited under, some of these methods will automatically download the given dataset, whereas others may require the manual download of the data. Users are pointed to the documentation of each method for instructions on how to properly load each dataset.

A full list of the datasets built into *RamanSPy* is available as part of the documentation of the package at https://ramanspy.readthedocs.io/en/latest/datasets.html.

METRICS

Users can likewise readily access relevant spectroscopic metrics, such as MSE, SAD and SID, from *ramanspy.metrics*. These can be used to measure the similarity between spectral data by using the respective methods, e.g.,:

```
ramanspy.metrics.SID(spectrum_I, spectrum_II)
```

A full list of the metrics built into *RamanSPy* is available as part of the documentation of the package at https://ramanspy.readthedocs.io/en/latest/metrics.html.

REFERENCES

[1] Harris, C.R.; Millman, K.J.; Van Der Walt, S.J.; Gommers, R.; Virtanen, P.; Cournapeau, D.; Wieser, E.; Taylor, J.; Berg, S.; Smith, N.J.; et al. Array programming with NumPy. *Nature* **2020**, 585, 357–362.
[2] The pandas development team. pandas-dev/pandas: Pandas. *Zenodo* **2020**; https://doi.org/10.5281/zenodo.3509134. Accessed 2 April 2020.
[3] Paszke, A.; Gross, S.; Massa, F.; Lerer, A.; Bradbury, J.; Chanan, G.; Killeen, T.; Lin, Z.; Gimelshein, N.; Antiga, L., et al. Pytorch: An imperative style, high-performance deep learning library. *Advances in neural information processing systems* **2019**, 32.
[4] Horgan, C. C.; Jensen, M.; Nagelkerke, A.; St-Pierre, J.-P.; Vercauteren, T.; Stevens, M. M.; Bergholt, M. S. High-throughput molecular imaging via deep-learning-enabled Raman spectroscopy. *ACS Analytical Chemistry* **2021**, 93, 15850-15860.
[5] Pedregosa, F.; Varoquaux, G.; Gramfort, A.; Michel, V.; Thirion, B.; Grisel, O.; Blondel, M.; Prettenhofer, P.; Weiss, R.; Dubourg, V.; et al. Scikit-learn: Machine learning in Python. *Journal of machine Learning research* **2011**, 12, 2825–2830.
[6] Ho, C.-S.; Jean, N.; Hogan, C. A.; Blackmon, L.; Jeffrey, S. S.; Holodniy, M.; Banaei, N.; Saleh, A. A.; Ermon, S.; Dionne, J. Rapid identification of pathogenic bacteria using Raman spec-troscopy and deep learning. *Nature communications* **2019**, 10, 1–8.
